# Assessments of the quality systems of pharmaceutical distributors: a remote approach to be applied in times of COVID-19 and beyond

**DOI:** 10.1186/s40545-021-00323-w

**Published:** 2021-05-10

**Authors:** Anthony Bourasseau, Laurine Lavergne, Raffaella Ravinetto

**Affiliations:** 1 Paris, France; 2 QUAMED, Brussels, Belgium; 3 Department of Public Health, Institute of Tropical Medicine, Nationalestraat 155, 2000 Antwerp, Belgium

**Keywords:** Quality of medicines, Pharmaceutical distributor, Supply chain, Remote audit, Distant assessment, Videoconferencing, Information technology, Travel restrictions, Developing countries, COVID-19

## Abstract

**Background:**

Adequate quality systems throughout pharmaceutical supply chains are crucial to protect individuals and communities from substandard and falsified medical products. Thus, pharmaceutical distributors are regularly assessed by qualified experts. Since the COVID-19 pandemic has forced a suspension of normal activities, remote assessments via videoconferencing may represent a temporary alternative to on-site audits. We exploratorily evaluated the feasibility of remote assessments of pharmaceutical distributors, located in a low- or middle-income country hard-to reach during the COVID-19 pandemic.

**Methods:**

We conducted pilot remote assessments of four conveniently selected distributors. The expert was remotely connected via videoconference, and supported by an in-country assessment facilitator (ICAF), who had received ad hoc training and was present at the assessed facility. First, the remote expert assessed the quality assurance (QA) activities and rated their compliance with the standards of the World Health Organization Good Storage & Distribution Practices (GSDP), as per routine practice. Second, s/he assessed the completeness, clarity and accuracy of data collected remotely, first per distributor, and then in aggregated form.

**Results:**

Data completeness was assessed by the expert as excellent, while clarity and accuracy were good. Overall data quality (a combination of completeness, clarity and accuracy) was good, with no major differences across QA activities, nor across distributors. Contextual limitations included poor internet connection, language barriers, and distributors’ lack of familiarity with QA terminology.

**Conclusions:**

Our findings are exploratory and cannot be extrapolated to other contexts, nor to other types of audits. Nonetheless, this pilot experience suggests that a well-planned remote assessment of pharmaceutical distributors, conducted with the support of a well-trained onsite ICAF, can provide data of acceptable quality, allowing to assess GSDP-compliance and to make temporary decisions about licensing or purchasing. Purchasers and policy makers should read the results of remote assessments in light of their intrinsic limitations. At the moment, onsite assessments remain the gold standards, but this could change in the longer term, with improved information technology and in light of the need to avoid unnecessary travels. Furthermore, remote assessments could be considered for routine pre-screening candidates for on-site assessments, and for targeted follow-up of on-site assessment.

**Supplementary Information:**

The online version contains supplementary material available at 10.1186/s40545-021-00323-w.

## Background

The Sustainable Development Goal 3.8 aims at universal health coverage (UHC), including quality and affordable essential medicines and vaccines for all. There is increasing evidence that adequate regulatory oversight and medicines quality assurance mechanisms throughout the supply chain are crucial to protect individuals and communities from substandard and falsified medical products, and that they are strictly interconnected with the goals of UHC [1]. Unfortunately, the quality systems of pharmaceutical distributors in low-income and middle-income countries (LMICs) tend to be weak [2], and insufficient to prevent and detect the supply of poor-quality medicines [3]. Furthermore, the weaknesses of National Regulatory Authorities (NRA) hinder the implementation of stringent regulatory supervisions along the supply chain in many LMICs. To correct this situation, the World Health Organization (WHO) started a Global Benchmarking of Regulatory Systems for evaluating the national regulatory systems through a comprehensive and systematic benchmarking [4, 5]. While achieving stringency of all NRAs remain the ultimate goal of stakeholders in pharmaceutical systems, short- and middle-term strategies are needed to secure the quality of medicines and other health products procured in poorly regulated contexts [6].

Twenty-six non-for-profit purchasers of medicines and other health products are currently members of QUAMED. QUAMED stands for ‘’Quality Medicine for All’’. It is a humanitarian alliance that aims to improve access to quality medicines, by raising awareness among key players in pharmaceutical supply systems, and by reinforcing the quality assurance and supply policies of its members [7]. Among its activities, QUAMED conducts assessments of pharmaceutical suppliers, and it advices its own members for the selection of reliable local suppliers. The assessments are conducted according to the standards of the WHO Good Manufacturing Practices (GMP) [8], for manufacturers of finished pharmaceutical products, of the WHO Model Quality Assurance System (MQAS) [9], for procurement agencies/distributors that prequalify their own sources and suppliers, and of the WHO Good Storage & Distribution Practices (GSDP) [10], for “technical visits” of distributors that do not prequalify their own sources and suppliers.

Pharmaceutical assessments traditionally require the on-site presence of a qualified expert, who carries out first-hand observations of policies, procedures, records and practices at the assessed facilities. Unfortunately, the COVID-19 pandemic has forced a partial suspension of normal activities, due to the restrictions on international travels. Under these circumstances, remote assessments could represent a temporary alternative to the on-site assessments. To the best of our knowledge, there are no explicit restrictions in the WHO and regulatory guidance to conduct remote audits or inspections, but there is no detailed guidance either. For the European Medicines Agency (EMA), a distant assessment is an “assessment of the compliance of a site (…) on the basis of documents and interviews and supported by technology for communicating, accessing systems, sharing and reviewing documents and other information, without the inspectors being physically present at the sites, where the activities subject to the assessment have taken place and where the inspection would ordinarily be hosted” [11]. According to EMA, “on-site inspections should be conducted when circumstances permit following the distant assessment”, and inspectors should make “a case-by-case decision on whether a distant assessment is considered appropriate and feasible. The criticality of the product should be taken into consideration”. Furthermore, the optimal communication platform could include a live videoconference platform with break-out rooms/conferences and screen sharing, smart glasses or other mobile cameras which can be interfaced to the videoconference platform, and access to a secure cloud server to share documents.”[11]. EMA also published a “Guidance on remote Good Clinical Practices inspections during the COVID-19 pandemic” [12], and the EU Medical Device Coordination Group published a detailed “Guidance on temporary extraordinary measures related to medical device Notified Body audits during COVID-19 quarantine orders and travel restrictions” [13]. For the United States Food and Drug Administration (US-FDA), a remote audit is an “audit performed off-site through the use of information and communication technology*”* (synonyms are eAudit, and virtual audit)[14]. At the beginning of the COVID-19 pandemic, the US-FDA postponed on-site GMP and GSDP inspections, before adjusting their strategy based on the risks [15]. The US-FDA’s Medical Device and Single Audit Program started a Remote Auditing pilot program, to gauge the viability of remote Device Marketing Authorization and Facility Registration Process [16], but to the best of our knowledge, no guidance has been issued for remote pharmaceutical inspections. Conversely, a few stakeholders in the private sector developed some guidance. For instance, Mark Durivage, from Quality Systems Compliance LLC, illustrated the pros and cons of remote GMP audits versus on-site audits [17], while Freyr Solutions linked remote “desktop” audits to the levels of risk in a self-speaking infographic [18].

Overall, even if video conferencing is today accepted practice in several sectors, there is little public information on the use of Information Technology (IT) and video-conferencing tools for the remote assessment of pharmaceutical facilities. In addition, scientific literature provides little or no guidance on whether this would be feasible. There is an urgent need to address this question, and to understand which factors could have an impact on the feasibility of remote pharmaceutical assessments, e.g., the nature of the audit, the characteristics of the audited facility, the quality of the in-country Internet connection, etc.

Therefore, this operational research was conducted to evaluate the feasibility of remote GSDP technical visits of pharmaceutical distributors located in an LMIC hard-to reach during the COVID-19 pandemic. Secondary objectives included describing the challenges, benefits and intrinsic limitations of the remote GSDP technical visits, adapting the QUAMED “on-site” GSDP procedure to the “remote” modality, and formulating recommendations on eligibility criteria, procedures, and tools for remote GSDP technical visits.

## Methods

### Study design, procedures and setting

**Fig. 1:**
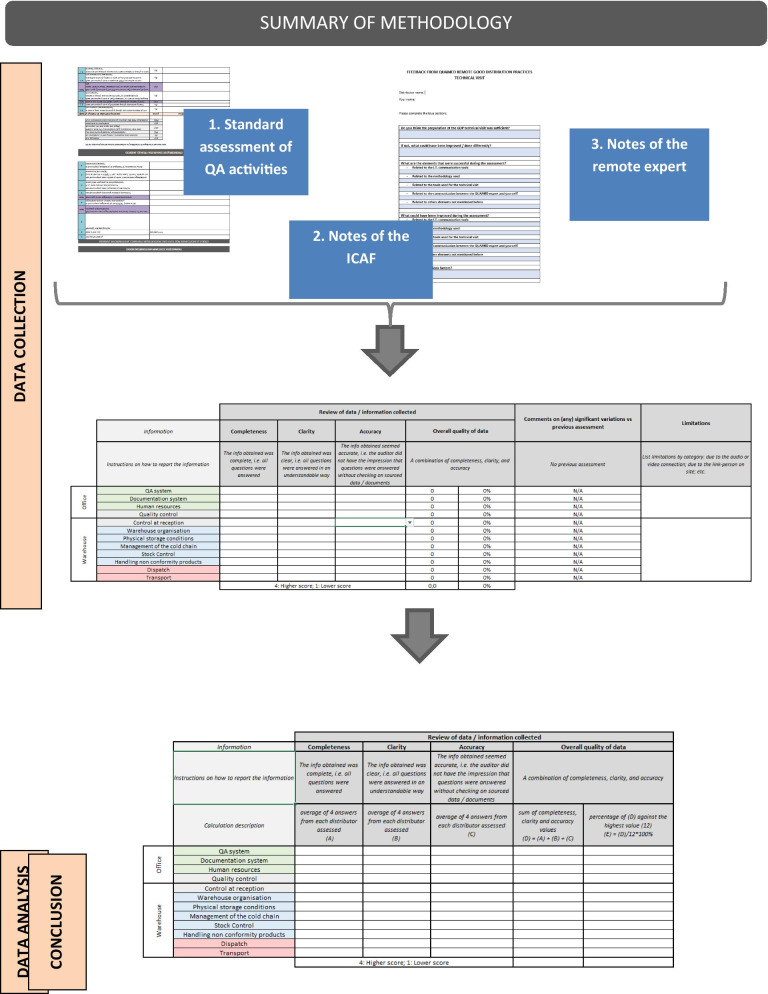
Summary of methodology

We conducted four pilot remote technical visits of pharmaceutical distributors, and assessed their feasibility. An overview of the study methodology is provided in Fig. 1.

### Development or adaptation of standard operating procedures (SOP)

We developed the SOP for the remote GSDP assessment. It is based on a previous SOP for on-site GSDP visit, which uses a standardized questionnaire, and a rating system that individually assesses twelve quality assurance activities [2]. The new SOP covers the respective roles and responsibilities of the remote expert and of the in-country assessment facilitator (ICAF), including confidentiality and conflict of interest, the practical setup for the remote technical assessment, including what should happen before it (e.g., sending upfront selected documents, including licenses, organigrams, list of supplied products, etc.), during it (e.g., interviews with key staff, visit of the premises, essential functions to be filmed or photographed etc.), and after it (i.e., the reporting aspects). Importantly, the SOP includes aspects specific to the remote methodology, such as the selection, minimum competencies and training of the in-country assessment facilitator (ICAF). The ICAF, who should be free from any conflict of interest, should be present at the assessed distributor for ensuring the link with the remote expert throughout the remote assessment, e.g., by positioning the video camera as required by the expert, doing translations, double checking on site documents etc.. We also adapted the SOP to address the use of digital technology (video and audio recording, pictures of premises, documents etc.) and the management of information obtained remotely, in compliance with the European Union’s General Data Protection Regulation 2016.

### Selection of country and distributors

The essential requirements for selecting the study country were the possibility to use IT communication, and the availability of support from QUAMED member organization(s) (from now onward called “QUAMED member”) working in-country, e.g., for selecting and contacting the distributors, appointing an ICAF etc. Countries where English or French are usually accepted working languages were preferred. Furthermore, in-country local distributors were eligible if indicated by a QUAMED member in the country as potential suppliers of medicines and health products.

A call for interest was sent to all QUAMED members in July 2020. The process resulted in the selection of an LMIC hard to reach during the pandemic, where two QUAMED members interested in this work were present, and of four pharmaceutical distributors within it. The four distributors accepted the QUAMED remote assessments. To keep the promise of confidentiality made to them, neither the country nor the distributors are made identifiable in the study report and in this manuscript.

### Selection of ICAFs

To be eligible as ICAF, local staff of QUAMED members operating in the study country needed to be fluent in English or French (depending on the country), to understand local languages (as applicable), and not to have any professional or other links to the assessed distributors. Importantly, they needed to have proven experience in medical supply chain management. Candidates with formal training in pharmacy or logistics, and/or previous knowledge of WHO GSDP were preferred. Potential ICAFs were identified by the QUAMED member(s) operating in the study country, and applications were assessed by the expert. Two experienced ICAFs were selected, a pharmacist, and a logistics manager trained in business administration, and remotely briefed by the expert. Each ICAF supported two out of four remote audits.

The remote assessment matrix is presented in Table 1.

**Table 1:** Remote assessment matrix

Country	Distributor	Linkage	Expert (remote)	ICAF (onsite)
Country X	Distributor 1	QUAMED member A	Expert 1	ICAF alpha
Country X	Distributor 2	QUAMED member B	Expert 1	ICAF beta
Country X	Distributor 3	QUAMED member A	Expert 1	ICAF alpha
Country X	Distributor 4	QUAMED member B	Expert 1	ICAF beta

### Data collection and analysis

The remote GSDP technical visits were conducted by the expert between 2nd and 23rd November 2020, with the expert remotely connected and one ICAF physically present onsite. Twelve standard QA activities were assessed by the expert, according to the usual procedure applicable for on-site visits, while the ICAF facilitated the expert’s visual access to the relevant spaces, processes and functions. Four out of 12 QA activities (i.e., assessment of the QA system, documentation system, human resources, and quality control) are mainly based on a desk assessment of the documentation provided by the distributor (i.e., office work), while the eight remaining can only be assessed by visiting (seeing) the premises (i.e., remote warehouse assessment). The expert rated each activity for GSDP-compliance. The product sourcing assessment, i.e., an evaluation by the expert of whether the products supplied by the distributors come from reliable manufacturers (based on GMP certification), was conducted by the expert according to the standard procedure as office work. The rating of the QA activities in terms of compliance with the WHO GSDP is separately reported in the QUAMED access-controlled database. The study-specific data were collected by the expert in a “data collection and analysis tool”, where the completeness, clarity and accuracy of data collected remotely for each of the 12 QA activities are rated. The information obtained for each activity was rated by the expert in terms of completeness, clarity and accuracy on a 0–4 scale, where a value above 3.5/4 corresponds to ‘excellent’, a value between 2.5–3.4/4, to ‘good’, and a value below 2.5/4, to ‘unsatisfactory’. The overall quality per activity, on a 0–12 scale resulting from the sum of completeness, clarity and accuracy, was assessed as ‘excellent’ if the overall value was above 10.5 out of 12, as ‘good’ if between 7.8 and 10.4/12, and ‘unsatisfactory’ if below 7.7/12. Data were further aggregated for the four distributors. Qualitative notes that helped the expert to formulate the ratings were collected in a “feedback forms” by the ICAFs, and in the notes taken by the expert during the assessment. The data collection and analysis tool is shown in Table 2.

### Ethics

The protocol was approved by the Institutional Review Board of the Institute of Tropical Medicine, Antwerp, Belgium (ref. 1422/20). Distributors freely accepted to be assessed, provided that their confidentiality was protected by not making them, nor the country, identifiable.

**Table 2:**
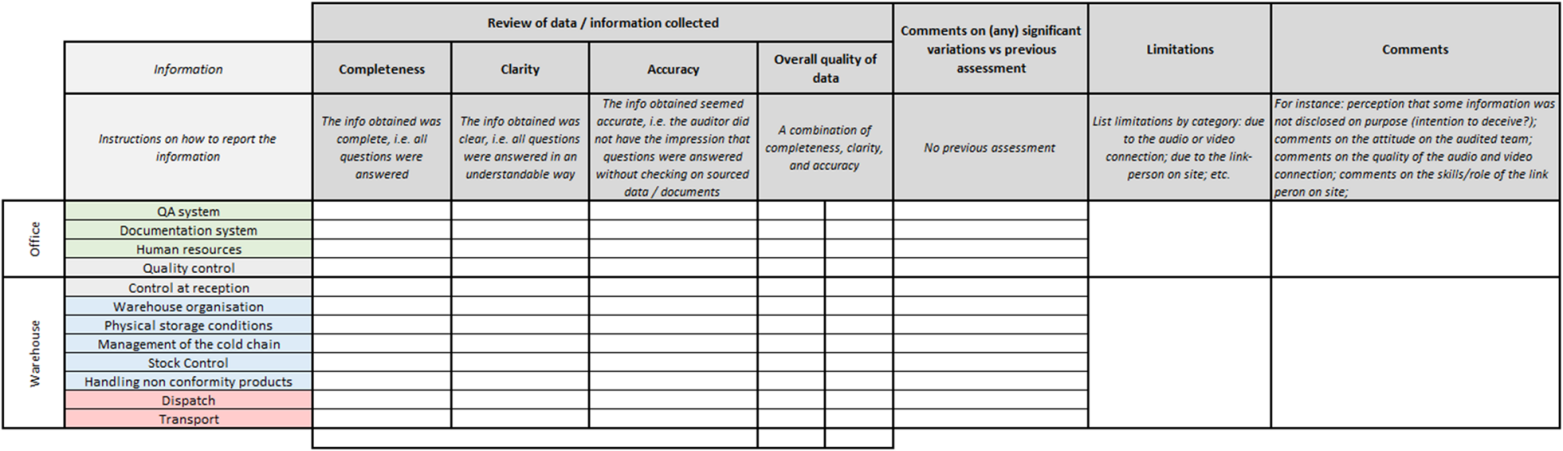
Data collection and analysis tool by distributor

## Results

Table 3 presents in aggregated form the results of the expert’s assessment of the completeness, clarity and accuracy of QA information obtained remotely for the four distributors. The completeness, i.e., in terms of questions that were answered in the standard QA questionnaire, was excellent (3.9 on a 0–4.0 scale), while the clarity, i.e., the understandability of the information, and the accuracy, i.e., the perceived reliability of the information, were good (3.1 and 3.2 on a 0–4.0 scale, respectively). The overall quality of data, calculated by summing completeness, clarity and accuracy, was good (10.2 on a 0–12.0 scale). As shown in Table 3, there was little variability across distributors.

**Table 3:**
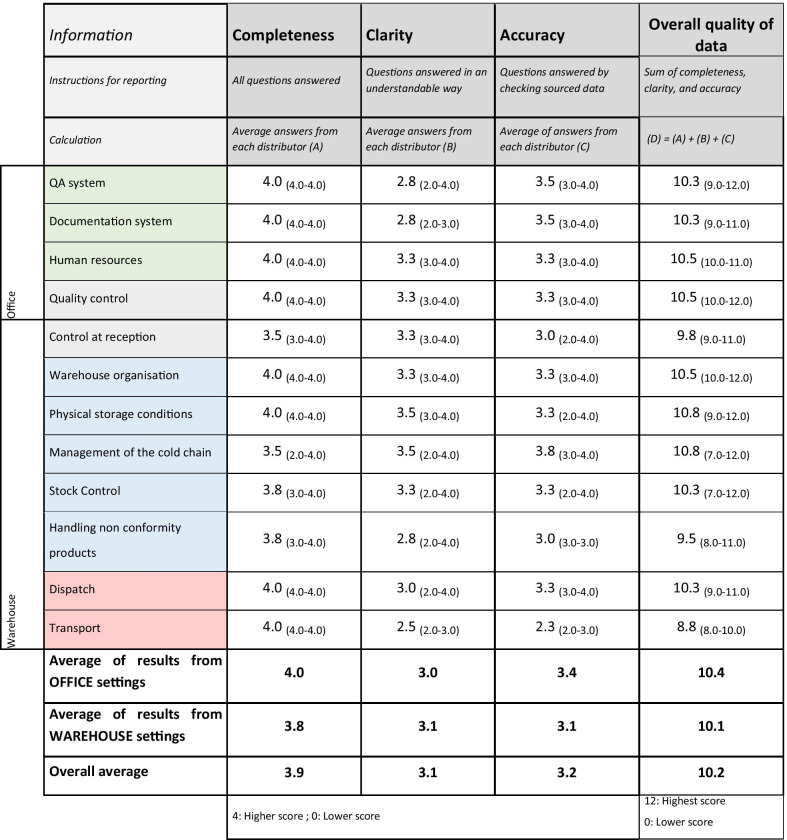
Completeness, clarity and accuracy of QA information obtained for GSDP remote assessments across the four distributors

**Fig. 2:**
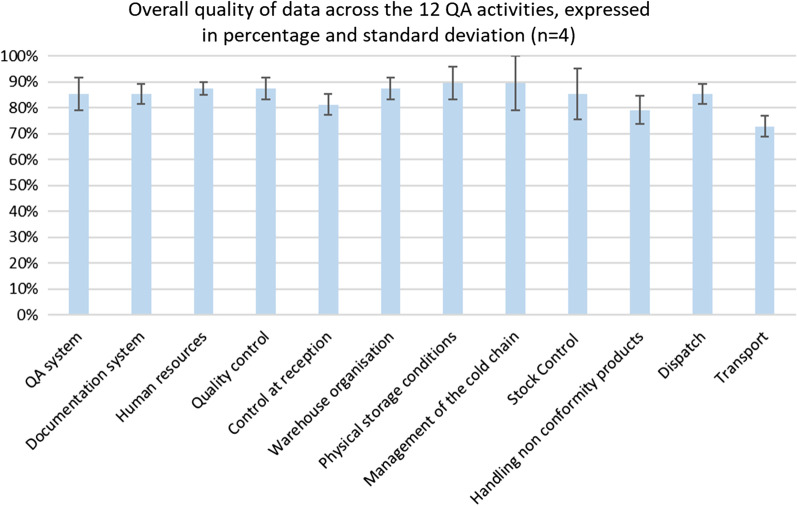
Overall quality (completeness, clarity, accuracy) of data obtained for the 12 QA activities

The difference between the quality of the information prevalently assessed as office work (i.e., desk assessment of documentation on the QA system, documentation system, human resources, and quality control) and the quality of the information assessed during the remote warehouse assessment (i.e., visit of the premises) was small (10.4 versus 10.1). When it comes to the quality of data collected for the 12 specific quality assurance activities, presented in Fig. 2, the lowest values (i.e., less than 10.0) are “handling non-conformity products”, “control at reception” and “transport”. When it comes to the product sourcing assessment, data provided by the distributors were complete, clear and accurate. This is not surprising, as the product lists provided by email are subject to a desk-assessment only, as it would happen for an on-site visit.

The observed contextual limitations include poor internet connection, particularly in warehouses, requiring frequent repetitions after audio or videoconferencing breaks, some language barriers, and some lack of familiarity in the study country with QA concepts and terminology. Noteworthy, both ICAFs expressed a strong interest to learn more on QA and to stay connected to the study group, after the study.

## Discussion

Some deviations were observed during the field work versus the planned procedures. In particular, we could not compare the findings of this remote audit to the findings of previous onsite audits (available for two out of four distributor), since different data collection tools had been used; the ICAF selection was not done by the QUAMED technical coordinator, but by the expert who would have worked with them, and the study-specific data on clarify, accuracy and completeness were not double checked by a second expert, because this would have lacked essential first-hand experience of the elements observed during the technical visits. Importantly, our findings are exploratory and cannot be extrapolated to other contexts, nor to other types of (more complex) audits (GMP, MQAS etc.). They should be confirmed in different contexts, with different levels of regulatory oversight, to check their replicability. During the next research phases, more tools, such as smart glasses or screen sharing features, could be tested. Furthermore, future research could use more sophisticated designs, for instance, by getting distributors assessed by two different experts in a cross-over sequence, by comparing results obtained at a same distributor with the support of different ICAFs, by adopting a mixed method design to triangulate data from different sources, and—importantly—by validating the methodology by means of comparison of findings from remote and on-site visits conducted at a same distributor.

Despite limitations, data remotely obtained for assessing the GSDP-compliance of 12 essential QA activities in our sample of four distributors was complete, clear and accurate. Variations in overall quality of data were small across distributors (range: 9.9–10.6), across activities (range: 8.8–10.8), and between data obtained for QA activities that require a desk assessment of documentation versus those purely based on the visit of the premises (10.1 versus 10.4). This pilot experience suggests that the intrinsic limitations of remote assessments, e.g., unstable internet connection (particularly in warehouses), languages barrier and impossibility for experts to personally verify premises and documents, can be mitigated by adopting adequate good practices. These include adequate audit preparation (e.g., list of documents to be assessed sent to the distributor at least 5 working days upfront, print-outs as well as adequate tools for videoconferencing should be prepared and tested upfront, etc.), ensuring that the ‘’expert and ICAF’’ pair has language skills adequate to the distributors’ context, and planning slightly more time than for an equivalent on-site visit. Furthermore, the role of ICAFs seems crucial for the performance of the remote assessment, as his/her capacity to guide and orient the remote expert will directly impact the assessment results. Therefore, other key components of the good practices are represented by the individualized training of ICAF, including the essential of GSDP, planning sufficient time and tools to collect the ICAF feedback on the audit, and ICAFs’ ability to act as translators as needed. It could also be helpful to have (smartphone) video filmed by a third person, which would allow the expert to see both the ICAF and the distributor representative, during the visit of the warehouse. A careful implementation of these good practices can ensure the feasibility and reliability of remote assessments, to be adopted when an onsite visit is not possible, either because of the pandemic, or of (low-intensity) conflicts, or other reasons.

Furthermore, the interest expressed by ICAFs to keep on working at QA, suggests that remote assessments can create the opportunity to raise awareness among (international and local) staff of organizations that procure medical products about the need to strengthen quality assurance systems, and they can be a starting point to build local capacities. Another potential advantage of the remote assessment is that it can allow to interlink the routine operational activity (assessments) with research activity, given that data collection and analysis can be better tracked and followed. Furthermore, if remote assessments were in the future validated as equivalent to the current gold standard, i.e., the on-site assessments, they would also have a positive environmental impact (we estimate that for this pilot study, there was an approximate savings of 1.2 tons of CO_2_ emission), they would allow financial savings from spared costs of accommodation, visa fees, per diem, and transport, and they would allow a rapid responsiveness to urgent needs, which can be relatively frequent particularly in humanitarian emergency settings.

For the time being, in addition to being temporarily used instead of on-site assessments when these are unfeasible, remote assessments could also be used as routine tools to complement and strengthen the on-site assessments. For instance, a remote assessment could be set up to pre-screen the distributors proposed for on-site assessment, and avoid unnecessary visits at suppliers which are of very poor quality, with additional savings. Furthermore, the remote methodology could be used for improving the auditor’s follow-up of the corrective action plan from onsite visits.

## Conclusion

Our exploratory findings suggest that in case of exceptional circumstances such as the COVID-19 pandemic, a remote assessment of pharmaceutical distributors conducted according to the identified good practices and with the support of a trained ICAF, can provide data of acceptable quality; allow to adequately assess GSDP compliance; and allow to make temporary decisions about licensing (regulators) or purchasing (international organizations, NGOs).

At the moment, on-site assessments remain the gold standards, so the use of remote assessments outside exceptional circumstances and for temporary decisions, would be conditional upon formal validation of this methodology. Meanwhile, the use of remote assessments could be separately considered as part of routine assessments, both for pre-screening candidates for on-site assessments, and for targeted follow-up of on-site assessment.

## Supplementary Information

**Additional file 1.** Study internal report (that contains authors’ identifiers).

## Data Availability

Deidentified source data are available in the Study Report, which is provided as Additional file 1.

## Abbreviations

DMA: Device marketing authorization
EC: European Commission
EEA: European economic area
EMA: European Medicines Agency
EU: European Union
FDA: Food and Drug Administration
GMP: Good manufacturing practices
GSDP: Good storage and distribution practices
ICAF: In-country assessment facilitator
IT: Information technology
ITM: Institute of Tropical Medicines, Antwerp
LMIC: Low- and middle-income country
MDCG: Medical Device Coordination Group
MDSAP: Medical Device and Single Audit Program
MQAS: Model of Quality Assurance System for procurement agencies
NRA: National Regulatory Authorities
QA: Quality assurance
QC: Quality control
QCP: Quality Certification Program
SOP: Standard operating procedure
UHC: Universal Health Coverage
USFDA: United States Food and Drug Administration
WHO: World Health Organisation
