# Supplementary material for: Assessments of the quality systems of pharmaceutical distributors: a remote approach to be applied in times of COVID-19 and beyond
Source: J Pharm Policy Pract. 2021 May 10;14:43. doi: 10.1186/s40545-021-00323-w (PMC8107414; doi:10.1186/s40545-021-00323-w)
Supplement: Supplementary file 1 — Additional file 1. Study internal report (that contains authors’ identifiers). [file 40545_2021_323_MOESM1_ESM.pdf]

Research study report

Evaluation of quality systems of medical product procurement centres and distributors: challenges and opportunities of remote GSDP technical visits and sourcing assessments in low- and middle-income countries hard-to reach during current COVID-19 pandemic

Principal investigator: *Anthony Bourasseau*
Pharmacist, independent researcher
Consultant to ITM

ITM co-Investigator: *Raffaella Ravinetto*
Public Health Department
Institute Tropical Medicine (ITM)
Antwerpen, Belgium

QUAMED co-investigator: *Laurine Lavergne*
Technical coordinator
QUAMED

Version: *3.0 (final)*
Date: *07.01.2021*

Table of contents

Table of contents 2
Acronyms 3
1. Introduction & background 4
2. Objectives of the study 7
3. Methods 7
   I. Study design 7
   II. Data collection 12
   III. Study limitations and deviations 13
   IV. Ethical issues 14
4. Results 15
   I. Data analysis of remote GSDP technical visit 15
   II. Overview of contextual limitations observed during the remote assessments 17
   III. Good practices needed for remote assessments 18
   IV. Feedbacks from ICAFs 18
   V. Data analysis of sourcing assessment 19
5. Discussion 19
6. Conclusion 21
7. Annexes 22
References 29

Acronyms

DMA	Device Marketing Authorization
EC	European Commission
EEA	European Economic Area
EMA	European Medicines Agency
EU	European Union
FDA	Food and Drug Administration
GMP	Good Manufacturing Practices
GSDP	Good Storage and Distribution Practices
ICAF	In-country assessment facilitator
IRB	Institutional Review Board (ITM)
IT	Information Technology
ITM	Institute of Tropical Medicines, Antwerp
ITM	Institute of Tropical Medicine of Antwerp
LMA	Local pharmaceutical Market Assessment
LMIC	low- and middle-income country
MDCG	Medical Device Coordination Group
MDSAP	Medical Device and Single Audit Program
MQAS	Model of Quality Assurance System for procurement agencies
NRA	National Regulatory Authorities
PI	Principal investigator
PQ	Prequalification
QA	Quality Assurance
QC	Quality Control
QCP	Quality Certification Program
QUATC	QUAMED Technical Coordinator
SOP	Standard Operating Procedure
UHC	Universal Health Coverage
USFDA	United States Food and Drug Administration
WHO	World Health Organisation

1. Introduction & background

The access to quality-assured medical products is crucial for access to health. As already stated in the background of previous research protocols jointly conducted by QUAMED and the Institute of Tropical Medicine of Antwerp (ITM), "*the Sustainable Development Goal 3.8 aims at universal health coverage, including "quality and affordable essential medicines and vaccines for all". But the increasing globalization of medical products production, coupled to the lack of resources of National Regulatory Authorities (NRA)*[i] *in most low- and middle-income countries (LMICs) make very difficult the implementation of stringent regulatory supervisions on the different steps along the supply chain (i.e. manufacturers, importers, procurement agencies, distributors, retail providers etc.). With porous supply chains, medical products with inadequate quality-assurance standards may reach patients or be procured by humanitarian or development medical programs funded by external donors*[ii]."

As a reminder, substandard (also called "out of specification") and falsified medical products are respectively defined by the World Health Organization (WHO) as: i) authorized medical products that fail to meet either their quality standards or specifications, or both and ii) medical products that deliberately/fraudulently misrepresent their identity, composition or source. All may go undetected, and result in avoidable morbidity, mortality, drug resistance.

QUAMED's vision and core activities

A number of nongovernmental organizations (NGOs) and other non-for-profit purchasers of medicines, being increasingly worried about the risk of purchasing non-quality assured medicines for their medical programmes in LMICs, joined forces and pooled resources in QUAMED, to work together for securing their pharmaceutical purchase. QUAMED was created in 2010 as a North-South network, with the aim to contribute to "improving access to quality medicines, by raising awareness among the key players involved in the pharmaceutical supply system and by reinforcing the quality assurance systems and supply policies of its members". Initially a project hosted in the Institute of Tropical Medicine of Antwerp, QUAMED evolved in 2017 into a non-for-profit organisation with the same vision, mandate and core activities, i.e. an humanitarian alliance for access to quality medicines (https://www.quamed.org/?lang=en).

Among its activities, QUAMED conducts: (a) audits to pharmaceutical manufacturers, (b) audits to public and non-for-profit procurement agencies, and (c) assessments of the local pharmaceutical market in the countries where its members work; and it give advice for the selection of reliable local suppliers. Audits and assessments are conducted in line with the standards of the WHO Model Quality Assurance System for procurement agencies (MQAS)[iii], WHO Good Storage & Distribution Practices (GSDP)[iv] and WHO Good Manufacturing Practices (GMP)[v], depending on the type of audited entity: WHO MQAS is used for procurement agencies/distributors that are also prequalifying their own sources and suppliers; WHO GSDP, for distributors that are not prequalifying their own sources and suppliers; and WHO GMP, for manufacturers of finished pharmaceutical products.

The assessment of the local pharmaceutical markets, called 'Local Market Assessments' (LMA), includes: (i) a rapid review of the national regulatory framework, (ii) GSDP/GMP technical visits (shorter than a MQAS audit) of selected local pharmaceutical distributors/manufacturers and (iii) product sourcing assessments (i.e., the verification that products supplied by audited suppliers are sourced based on adequate quality standards and specifications). Reports and related information obtained from these assessments are kept in an access-controlled database, only available to QUAMED members.

The methodology and tools used by QUAMED require the on-site presence of a qualified expert. Unfortunately, the COVID-19 pandemic has forced a suspension of normal activities, due to the restrictions on travels. Nonetheless, the need of up-to-date and reliable information on the quality of medical products is not only still present, but even magnified (at least in some countries), due to the difficulties to procure medicines through the usual suppliers and the consequent need to rapidly assess new potential suppliers.

Remote GSDP audit/assessment of pharmaceutical procurement agencies/distributors: challenges, risks and opportunities in COVID-19 pandemic

On-site audits and assessments are and remain the gold standard, because they allow first-hand observations of policies, procedures, records and practices as adopted and implemented at pharmaceutical facilities. Nevertheless, there are no restrictions in the WHO GMP/GSDP regulations, standards or other guidance documents to conduct remote audits and assessments; but unfortunately, there are no stringent international guidelines for such situations either.

Video conferencing, including the use of screen-sharing and other Information Technology (IT) advancements, are today accepted practices in several fields of activity, so it could be assumed that a remote assessment (equivalent as a distant assessment) could be as efficient and effective as one performed onsite, provided that it can be supported by the adequate video-conferencing technology. However, whether the use of these advanced technologies would allow the same level of quality as in an on-site assessment, depends on several other factors, including but not limited to the nature of the audit/assessment, the structure of the audited/visited facility, the skills of the auditor/assessor (acting remotely) and of his/her on-site counterpart, the quality of the Internet connection, the possibility of sharing documents and records etc.

When considering whether to do a remote audit/assessment, we should carefully balance the benefits and the risks in a specific context, for a specific supplier, and for the specific kind of medical products supplied. Such benefits and risks should also be carefully weighed versus those of the gold standard, that is the on-site audit. Nonetheless, there are situations when an on-site audit is not possible – as it is currently the case in many countries, due to COVID-19-related travel restrictions; but it can also be the case elsewhere, for instance in some conflict areas. Therefore, an alternative to the gold standard is needed, and it should be chosen based on a careful and contextualized risk assessment.

Definition of remote or distant inspection / audit / assessment

For the European Medicines Agency (EMA), "a distant assessment" is defined as an "*Assessment of the compliance of a site (…) on the basis of documents and interviews and supported by technology for communicating, accessing systems, sharing and reviewing documents and other information, without the inspectors being physically present at the sites where the activities subject to the assessment have taken place and where the inspection would ordinarily be hosted*" [vi].

For the United States Food and Drug Administration (USFDA), a remote audit is defined as *"audit performed off-site through the use of information and communication technology"* (synonyms are eAudit, and virtual audit) [vii].

Practices by major regulators

Remote audits/assessments present challenges and opportunities, with which major regulators and other stakeholders have been struggling with, just as QUAMED today.

<u>European Commission (EC) / European Medicines Agency</u>

In a Guidance published in October 2020, EMA describes how distant GSDP/GMP assessment could be carried out when on-site assessment is not feasible[vi]. The guidance highlights that *"on-site inspections should be conducted when circumstances permit following the distant assessment"* and that inspectors should make *"a **case-by-case decision** on whether a distant assessment is considered appropriate and feasible. The **criticality of the product** should be taken into consideration."*. For EMA, *"the optimal communication platform could include the following:*

- *A live videoconference platform which has the following capabilities:*
    - *Break-out rooms/conferences to facilitate separate channels of discussion*
    - *screen sharing to display site applications/electronic systems.*

- *Smart glasses or other mobile cameras which can be interfaced to the videoconference platform to provide live footage of manufacturing operations, facilities and equipment.*
- *Access to a secure cloud server to share documents."*

Furthermore, EMA had published in May 2020 a *"Guidance on remote Good Clinical Practices inspections during the COVID19 pandemic"*[viii].

The EU Medical Device Coordination Group (MDCG), published in 2020 a "*Guidance on temporary extraordinary measures related to medical device Notified Body audits during COVID-19 quarantine orders and travel restrictions"*[ix] where eligibility criteria and procedural aspects are well-detailed.

United States Food and Drug Administration (USFDA)

The US FDA decided to cancel on-site GMP and GSDP inspections for the duration of the COVID-19 outbreak, but no document was found (as to 22/12/2020) that describes the use of remote inspections for pharmaceutical products. The situation is different for medical devices, where performing remote inspections in certain conditions is accepted by the USFDA: the FDA's Medical Device and Single Audit Program (MDSAP), in particular, launched a Remote Auditing pilot program to gauge the viability of remote Device Marketing Authorization (DMA) and Facility Registration Process[x].

Practices in the private sector

Selected articles or documents published by stakeholders in the private sector in specialised fora provide some guidance on how to run a remote audit. For instance, Mark Durivage, from Quality Systems Compliance LLC, illustrated the pros and cons of remote GMP audits *versus* on-site audits[xi]. These elements can easily be extrapolated to GSDP audits:

Advantages of On-site Audits	Advantages of Remote Audits
• Enhanced ability to visualize the facility, equipment, and processes<br>• Records are easier to produce<br>• Easier to adapt to what is observed<br>• Better ability to read body language	• No travel costs<br>• No lost time due to travel<br>• More flexible scheduling<br>• Minimize the risk of contamination by the auditor (i.e. COVID-19)

Table 1: Advantages of on-site audits versus remote audits

Freyr solutions, a consulting company, linked remote "desktop" audits to the levels of risk in a self-speaking infographic[xii]. It should be noted that this schematic approach may not be feasible when travels are prohibited.

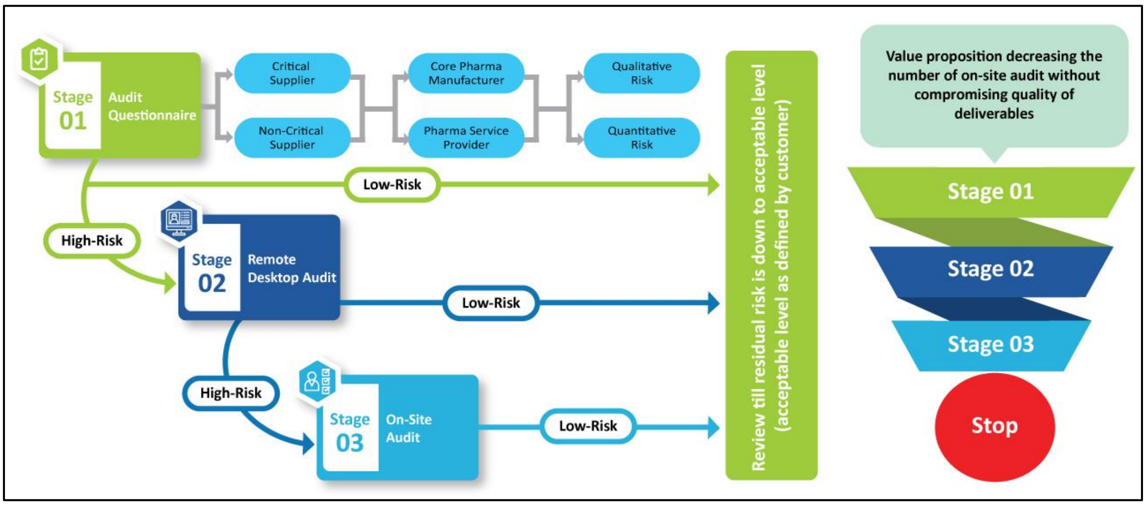

Figure 1. Type of audits linked with level of risks (Freyr solutions)

This research

The research, presented in the protocol *"Evaluation of quality systems of medical product procurement centres and distributors: challenges and opportunities of remote GSDP technical visits and sourcing assessments in low- and middle-income countries hard-to reach during current COVID-19 pandemic",* was written in collaboration between QUAMED and the ITM, in the frame of a research collaboration in place between them. The protocol was submitted to the ITM Institutional Review Board (IRB) on 14th July 2020. A conditional approval was received on 06th August 2020, with two comments. The protocol modified as requested in the conditional approval was notified on 03th November 2020 to the IRB. The protocol is attached in **Annex 1**.

Why choosing remote GSDP technical visit for this feasibility study?

QUAMED has gained over the years a significant experience and expertise in local market assessments (LMA), which include GSDP technical visits, and sometimes GMP technical visits. QUAMED members are continuously requesting LMA from QUAMED, more than any others services. This is the reason why GSDP technical visits were chosen for this feasibility study.

2. Objectives of the study

Main objective

- To evaluate the feasibility of (i) remote GSDP technical visits of pharmaceutical suppliers located in a LMIC hard-to reach during the current COVID-19 pandemic and (ii) of remote product sourcing assessments. Such "remote activities" consist of a combination of virtual activities by a qualified expert located outside the country, and on-site activities by a selected on-site third person.
- To describe the risks and benefits of remote GSDP technical visits and sourcing assessments.

Specific objectives

1. To adapt the current "on-site" GSDP technical visits and sourcing assessment procedure, for "remote" GSDP technical visits and sourcing assessments, i.e. conducted via a combination of remote and on-site activities;
2. To pilot the methodology for the remote GSDP technical visits and sourcing assessments in at least one LMIC hard-to reach during the current COVID-19 pandemic;
3. To describe the risks, benefits and limitations of the remote GSDP technical visits and sourcing assessments;
4. To formulate recommendations on procedures, tools and criteria of eligibility for remote GSDP technical visits and sourcing assessments.

3. Methods

I. Study design

- **Development and approval of standard operating procedures and tools in the QUAMED quality management system**

First, the PI conducted a gap analysis on the QUAMED quality management system, to assess if it was ready for supporting the conduct of remote assessments. This initial evaluation indicated that three types of documentation were partially or fully lacking:

  - Specific procedures and tools related to remote GSDP technical visits and sourcing assessment

  - A procedure related to the management of confidentiality and conflicts of interest for non-QUAMED staff (such as field staff of QUAMED members) involved in a technical visit;
  - A procedure related to the data management of data collected remotely.

Second, the “missing” SOP were developed.

- The new SOP for the remote GSDP technical visit and sourcing assessment (QUAMED no. SOP-Q-021) is based on the Local Market Assessment SOP (QUAMED no. SOP-Q-019), but it specifies elements such as : (i) responsibilities assigned; (ii) selection and minimum competencies of in-country assessment facilitator (ICAF), (iii) potential needs of ICAF training; (iv) management of confidentiality and conflicts of interest for all stakeholders; (v) practical set-up for the remote technical visits and (vi) reporting aspects. This tool allows a standardised approach using standard questions and a corresponding rating system, leading to an overall GSDP rating score for (a) each distributor, and (b) each of the 12 quality assurance activities.
  The “tool” annexed to this SOP (**Annex 2**: ‘*Distributor GSDP technical visit & product source assessment tool’*) was also adapted, to include some practical elements to be used during the remote assessment. (It should be noted that the assessment tool for on-site GSDP technical visits had been revised in 2020, and the new version was not used in real settings prior to the remote GSDP technical visits).

- The development of an ad hoc procedure for the management of confidentiality and conflict of interest was deemed important, as the previous system linked these elements with the QUAMED auditor contract. In the situation of remote assessment, however, both the QUAMED quality assurance expert (i.e. the auditor) and the local third part (i.e., a NGO staff) should sign the form. The QUAMED SOP no. SOP-G-001 (Confidentiality, code of conduct and Conflicts of interest) covers these elements.

- Lastly, the remote assessments require a lot use of digital technology. Thus, ad hoc measures should be in place to ensure that data collected remotely (video and audio recording, pictures of premises, documents etc) are managed in such a way to comply with European Union’s General Data Protection Regulation (GDPR, 2016) and to protect key information shared from the distributors assessed. QUAMED SOP no. SOP-Q-007 (Handling information within QUAMED network) was updated to cover these aspects.

These new or revised SOPs have been integrated into the QUAMED quality management system, reviewed by Technical Committee members and approved by QUAMED technical coordinator.

◎ Selection process of country and distributors

Note: In this report, ‘country’, ‘distributor name’, ‘NGO name’ and ‘ICAF name’ have been anonymised to ensure the confidentiality.

The selection criteria (as set in the QUAMED SOP no. SOP-Q-021) for the couple country / distributor were as follows:

- *Proposal of a QUAMED partner, based on current needs*
- *In-country support available, from a QUAMED partner*
- *Relevant product range*
- *English-speaking or French-speaking countries preferred*
- *Possibility to use IT communication tools for the remote technical visit.*

A call to participate to this pilot study was sent to all QUAMED members by the QUAMED director in July 2020. Three NGOs replied to the call. A rapid standardized survey across them allowed to identify targeted countries and distributors. One NGO only expressed interest in a country with limited English or French speaking

counterparts, and was not further selected. The two other QUAMED members identified the same country as first option, with four different distributors as their 3 primary choices.

SUMMARY OF METHODOLOGY

DATA COLLECTION

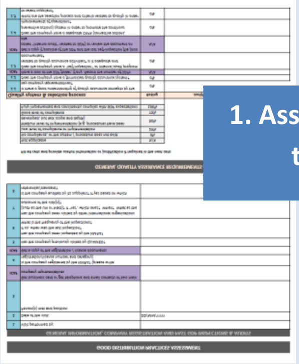

1. Assessment tool

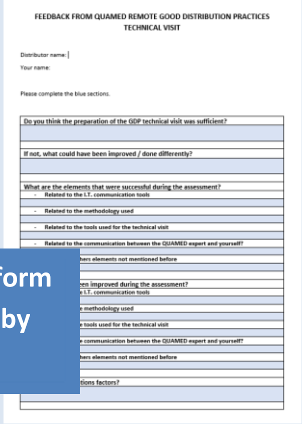

2. Feedback form completed by ICAF

3. Personal notes during remote technical visits from the principal investigator

	Information	Completeness	Clarity	Accuracy	Overall quality of data		Comments on (any) significant variations vs previous assessment	Limitations
		Review of data / information collected						
	Instructions on how to report the information	*The info obtained was complete, i.e. all questions were answered*	*The info obtained was clear, i.e. all questions were answered in an understandable way*	*The info obtained seemed accurate, i.e. the auditor did not have the impression that questions were answered without checking on sourced data / documents*	*A combination of completeness, clarity, and accuracy*		*No previous assessment*	*List limitations by category: due to the audio or video connection; due to the link-person on site; etc.*
Office	QA system				0	0%	N/A	
	Documentation system				0	0%	N/A	
	Human resources				0	0%	N/A	
	Quality control				0	0%	N/A	
Warehouse	Control at reception				0	0%	N/A	
	Warehouse organisation				0	0%	N/A	
	Physical storage conditions				0	0%	N/A	
	Management of the cold chain				0	0%	N/A	
	Stock Control				0	0%	N/A	
	Handling non conformity products				0	0%	N/A	
	Dispatch				0	0%	N/A	
	Transport				0	0%	N/A	
		4: Higher score; 1: Lower score			0,0	0%		

DATA ANALYSIS

	Information	Completeness	Clarity	Accuracy	Overall quality of data	
		Review of data / information collected				
	Instructions on how to report the information	*The info obtained was complete, i.e. all questions were answered*	*The info obtained was clear, i.e. all questions were answered in an understandable way*	*The info obtained seemed accurate, i.e. the auditor did not have the impression that questions were answered without checking on sourced data / documents*	*A combination of completeness, clarity, and accuracy*	
	Calculation description	*average of 4 answers from each distributor assessed (A)*	*average of 4 answers from each distributor assessed (B)*	*average of 4 answers from each distributor assessed (C)*	*sum of completeness, clarity and accuracy values (D) = (A) + (B) + (C)*	*percentage of (D) against the highest value (12) (E) = (D)/12*100%*
Office	QA system					
	Documentation system					
	Human resources					
	Quality control					
Warehouse	Control at reception					
	Warehouse organisation					
	Physical storage conditions					
	Management of the cold chain					
	Stock Control					
	Handling non conformity products					
	Dispatch					
	Transport					
		4: Higher score; 1: Lower score				

CONCLUSION

Results of the feasibility study

<u>Country selection</u>

	NGO A	NGO B	NGO C
Country X	**Key criteria met**	**Key criteria met**	
Country Z			**Key criteria not met**

Table 2. Country selection

⇨ The country X was selected, with two QUAMED members (NGO A & B) supporting the pilot study.

<u>Distributors selection</u>

Distributor selected	NGO A		NGO B	
First choice	Distributor 1	**Key criteria met**	Distributor 1	**Key criteria met**
Second choice	Distributor 2	**Key criteria met**	Distributor 2	**Key criteria met**
Third choice	Distributor3	**Key criteria met**	Distributor4	**Key criteria met**

Table 3. Distributor selection

⇨ The distributor 1, 2, 3 & 4 were finally selected.

In order to keep the confidentiality promise, the country as well as the identity of the visited distributors are not disclosed in this report, in the following scientific article and in any other public documents.

◎ **Selection process of ICAFs**

The SOP no. SOP-Q-021 on "Conduct of QUAMED remote GSDP technical visit and product source assessment" lists some general, minimum requirements related to the selection of ICAFs:

- *"Languages: French and/or English according to the country of assessment; local language (if any) highly recommended to ease the communication*
- *Experience in medical supply chain management*
- *Diploma in pharmacy or logistics preferred.*
- *Good knowledge of WHO Good Distribution Practices."*

These criteria were transmitted to the QUAMED members headquarter counterparts, who selected the best candidate and shared their CVs with the QUAMED technical coordinator and the PI. Interviews were conducted by the PI using videoconferencing tool. The final decision was done by the PI alone, with no involvement of the QUAMED technical coordinator (refer to the section on "Study limitations and variations").

Criteria	ICAF alpha (NGA A)	ICAF beta (NGO B)
Education	Pharmacy	Business administration
Language	Good level of English<br>Local languages: Native or fluent	Good level of English<br>Local languages: Native or fluent
Current job	Pharmacy officer (in charge of stock management and supply)	Deputy logistics manager (in charge of procurement and supply chain management of health products)
Number of years of professional experience	Approximately 8 years	More than 10 years
Knowledge of WHO good distribution practices	Good	Limited
Selection	**Key criteria met**	**Key criteria met,** but the training / informative briefing should focus on the fundamentals of GSDP

Table 4. Selection of ICAFs

Note: The 2 QUAMED members expressed their will to have additionally a "country focal point", acting as coordinator between QUAMED, the headquarter staff and the ICAF. The selection process of these two persons (from the country/project management team) was internal to the NGOs, with no involvement of the PI.

- **Briefing and additional training of ICAFs**

After selection, the QUAMED tool for remote GSDP technical visits was sent to the ICAFs, as well as the latest version of WHO's Good Storage & Distribution Practices guidelines. The ICAF with limited knowledge on GSDP was requested to read the guidelines upfront. Then, a virtual briefing was held with the two ICAFs to go through the QUAMED remote GSDP assessment tools and processes, and to present their role.

- **Final remote assessments matrix**

Country	Distributor	NGO	ICAF
Country X	Distributor 1	NGO A	ICAF alpha
Country X	Distributor 2	NGO B	ICAF beta
Country X	Distributor 3	NGO A	ICAF alpha
Country X	Distributor 4	NGO B	ICAF beta

Table 5. Final remote assessment matrix

- **Product sourcing assessment**

The sourcing assessment requires the completion of the following table, which is included in the GSDP technical visit template (Figure 2):

PHARMACEUTICAL SOURCE COLLECTION AND EVALUATION

Sources Level 5	Manufacturing site GMP approved by the authorities of a country with Stringent Inspectorate Body (please refer to www.quamed.org for more details)
Source level 4	Manufacturing site GMP approved by a reliable but unofficial entity (QUAMED, MSF, UNICEF, ICRC...) after a full GMP audit.
Source level 3	Manufacturing sites with a satisfactory technical visit result (QUAMED experts) or an intermediate NRA approval (please refer to www.quamed.org for more details)
Source level 2	Manufacturing sites with an average technical visit result (QUAMED experts)
Source level 1	Manufacturing sites with proven unsatisfactory GMP compliance level.
No information	Absence of reliable information.

TO BE COMPLETED BY GDP TECHNICAL VISIT EXPERT			TO BE COMPLETED BY QUAMED HEADQUARTER TEAM		
Manufacturer	Address /Site	Country	GMP category	Source of information	Comments

Figure 2. Extract of the GDSP technical visit template showing the product sourcing assessment

First, the expert completes the first part of the table, based on the Product List provided by the audited distributors. Then, the QUAMED technical coordinator completes the second part of the table, based on the quality information (i.e., GMP level) available in the QUAMED database on each of the products included in the distributors' lists. The procedure used during on-site visits and remote visits is identical: product lists were transmitted by email by the ICAF or the distributor, and the same tasks were assigned to the QUAMED expert and the QUAMED technical coordinator.

- **Visits preparation and planning**

As per QUAMED SOP no. SOP-Q-021, the distributors' management teams were initially contacted by phone by the ICAFs and/or the NGOs country focal points. Then, a formal letter was sent to them, detailing the purposes and key-aspects of remote assessments, and the confidentiality clauses. The four distributors accepted the QUAMED technical visits.

- **Conduct of the remote GSDP technical visits and sourcing assessments**

The remote GSDP technical visits were conducted between 2nd to 23rd November 2020.

II. Data collection

◎ Non-study specific data

The expert/PI collected in first place the data for the assessment itself. These are the same that would be collected during an on-site technical visit, to assess the compliance with each of the relevant GSDP-activities. They are not study-specific, and are reported internally according to the usual QUAMED procedures in (i) individual remote GSDP technical report for each distributor, and (ii) a remote local market assessment report.

For distributors that had been previously assessed on-site by QUAMED, the data collected were triangulated with retrospective data from previous on-site assessments. These were available in the access-controlled QUAMED database which contains the results and reports of LMA conducted by QUAMED experts. Raw data from retrospective on-site assessments were confidentially shared with the PI for the scope of this work, but will remain under exclusive ownership of QUAMED, as well as the new raw data generated during this work.

◎ Study-specific data

The raw data were collected in (i) the study-specific "remote GSDP technical visit questionnaire" filled in by the auditor/PI, for reporting on completeness, clarity and accuracy of data collected remotely; (ii) the "feedback forms" filled in by the ICAFs and (iii) the "notes" taken by the PI during the assessment. For point (i), an ad hoc data collection and analysis tool was used, with two sections:

- A section with individual data from each distributor (Table 6):

		Review of data / information collected						Comments on (any) significant variations vs previous assessment	Limitations	Comments
	Information	Completeness	Clarity	Accuracy	Overall quality of data			
	Instructions on how to report the information	*The info obtained was complete, i.e. all questions were answered*	*The info obtained was clear, i.e. all questions were answered in an understandable way*	*The info obtained seemed accurate, i.e. the auditor did not have the impression that questions were answered without checking on sourced data / documents*	*A combination of completeness, clarity, and accuracy*	*No previous assessment*	*List limitations by category: due to the audio or video connection; due to the link-person on site; etc.*	*For instance: perception that some information was not disclosed on purpose (intention to deceive?); comments on the attitude on the audited team; comments on the quality of the audio and video connection; comments on the skills/role of the link peron on site;*
Office	QA system							
	Documentation system							
	Human resources							
	Quality control							
Warehouse	Control at reception							
	Warehouse organisation							
	Physical storage conditions							
	Management of the cold chain							
	Stock Control							
	Handling non conformity products							
	Dispatch							
	Transport							

Table 6. Data collection and analysis tool (section with data from each distributor)

- A section with aggregated data from all distributors (Table 7):

		Review of data / information collected				
	Information	Completeness	Clarity	Accuracy	Overall quality of data	
	Instructions on how to report the information	*The info obtained was complete, i.e. all questions were answered*	*The info obtained was clear, i.e. all questions were answered in an understandable way*	*The info obtained seemed accurate, i.e. the auditor did not have the impression that questions were answered without checking on sourced data / documents*	*A combination of completeness, clarity, and accuracy*	
	Calculation description	*average of 4 answers from each distributor assessed (A)*	*average of 4 answers from each distributor assessed (B)*	*average of 4 answers from each distributor assessed (C)*	*sum of completeness, clarity and accuracy values (D) = (A) + (B) + (C)*	*percentage of (D) against the highest value (12) (E) = (D)/12*100%*
Office	QA system					
	Documentation system					
	Human resources					
	Quality control					
Warehouse	Control at reception					
	Warehouse organisation					
	Physical storage conditions					
	Management of the cold chain					
	Stock Control					
	Handling non conformity products					
	Dispatch					
	Transport					
		4: Higher score; 1: Lower score				

Table 7. Data collection and analysis tool (section with aggregated data from all distributors)

Regarding the *product sourcing assessment* (i.e., the verification that products supplied by audited suppliers are sourced based on adequate quality standards and specifications), we did not develop specific "remote" tools, as the data collection and data analysis was carried out from the data generated in the technical report. Product sourcing assessments are always done by desk review, even for on-site technical visits.

III. Study limitations and deviations

◎ Variations between the study protocol and the methodology used in real settings

Data verification during collection and analysis

According to the protocol, *"the information from 2. and 3.*[1] *will be double checked by the QUAMED co-investigator, and if necessary, in case of discordant opinion, a third opinion will be sought by a QUAMED technical committee's representative."* This could not be done as planned, because the rating and information in the data collection and analysis tool were based on the actual technical reports' information, *but also* on the elements *observed* during the technical visits. The latter are not available to the QUAMED technical committee's representative, who did not participate in the remote technical visits.

Comparison of findings from e prospective remote assessments vs findings of previous on-site assessments

It was not possible to fully compare the results of the previous QUAMED on-site assessments (available for two distributors) due to the following limitations: (i) the GSDP remote technical visit assessment tool has been upgraded between the onsite and remote assessments, not allowing for direct comparability; (ii) the collection tool developed and used for this study was not filled in during the past on-site assessment, and completing it *a posteriori* using the on-site assessment report would bring inherent errors.

◎ Application of the pilot SOP in real settings: main deviations

The pilot SOP no. SOP-Q-021 ("Conduct of QUAMED remote GSDP technical visit") was written based on a theorical frame. The large majority of the elements included in this SOP were applicable in the pilot phase, but some deviations have also been observed when piloting the SOP requirements in this research:

What the pilot SOP says	What was actually done
QUAMED and the leading members should get written authorization of the company to use IT communication tools, take pictures etc.	A letter was sent to all distributors by email; 3 responded positively, but one did not respond in written. At the beginning of all technical visits, the auditor/PI asked again the distributor representatives' consent to use video conferencing tool and to take pictures.
Final selection and validation of the ICAF are carried out by QUAMED Technical Coordinator (QUATC).	The selection and validation of the ICAF was performed fully by the QUAMED auditor. This is considered adequate, as it is him/her who carried out the interviews with the ICAFs.
The QUATC or the Field Focal Point should send to the company-ies to be visited the list of documentation that may be requested during the GSDP technical visit	This was done for 3 out of 4 distributors by the auditor/PI, and not by the QUATC or the Field Focal Point. Due to time constraints, this could not be done upfront for the fourth distributor.

[1] "1. For each of the 13 assessed activities, the PI will describe (a) whether the information obtained via the remote assessment was clear and complete enough to draw conclusions; (b) if not, what aspects of the available information were unclear or incomplete; and (c) why; 2. For each distributor, the PI will further describe and discuss the potential determinants of observed shortcomings in the quality and completeness of the information collected via the remote assessment: e.g. adequacy of procedure and tools, third-person's qualification (pharmacist vs logistician, etc), quality of the Internet connection, transparency and collaborative approach of the staff of the audited distributor, etc". Extract from the research protocol.

Table 8. Main variations between the SOP content and the implementation in the field

The pilot SOP no. SOP-Q-021 will now be revised based on the findings of this pilot experience, upon approval of the study report.

IV. Ethical issues

The protocol was submitted for approval to the ITM Institutional Review Board on 14th July 2020. It was approved under the conditionality of the provision of further information on these two points:

- Selection procedure for the 'the "in-country assessment facilitator" (especially with regards to the management of confidentiality and conflict of interest)
- Digital security of the used platforms.

These two points have been covered in the QUAMED SOPs. The protocol modified as requested in the conditional approval was notified to the IRB on 3rd November 2020.

Prior to starting the GSDP technical visits and sourcing assessments, the management of each pharmaceutical distributor was informed of the purpose of the technical visit by QUAMED by phone and by email. Distributors were only assessed if they freely accepted to be assessed, and allowed the in-country assessment facilitator to access their facilities. We guarantee their confidentiality by not making the distributors identifiable neither in this analysis nor in any subsequent publication. Neither the name of the distributors or other possible identifiers, including the country name(s), will appear anywhere – unless explicitly requested by the concerned people.

Each in-country assessment facilitator and field focal point, as well as the QUAMED expert/PI, signed a confidentiality agreement and a conflict-of-interest form. No conflict of interest was identified.

4. Results

Note: *The expert/PI collected in first place the data for the assessment itself, which are not part of the study, and are reported internally in QUAMED. These data are included in (i) individual remote GSDP technical report for each distributor, and (ii) a remote local market assessment report.*

I. Data analysis of remote GSDP technical visit

◎ Completeness, clarity and accuracy

Table (Table 9) presents the auditor/PI assessment of the completeness, clarity and accuracy of QA information obtained for the four distributors, during the remote GSDP assessments (and reported in the standardized QUAMED technical visit reports).

Data are presented here (Table 9) in aggregated form, while individual results by distributors are found in **Annex 3**:

		Review of data / information collected				
	Information	**Completeness**	**Clarity**	**Accuracy**	**Overall quality of data**	
	Instructions on how to report the information	*The info obtained was complete, i.e. all questions were answered*	*The info obtained was clear, i.e. all questions were answered in an understandable way*	*The info obtained seemed accurate, i.e. the auditor did not have the impression that questions were answered without checking on sourced data / documents*	*A combination (the sum of completeness, clarity, and accuracy*	
	Calculation description	*average of 4 answers from each distributor assessed (A)*	*average of 4 answers from each distributor assessed (B)*	*average of 4 answers from each distributor assessed (C)*	*sum of completeness, clarity and accuracy values (D) = (A) + (B) + (C)*	*percentage of (D) against the highest value (12) (E) = (D)/12*100%*
Office	QA system	4.0	2.8	3.5	10.3	85%
Office	Documentation system	4.0	2.8	3.5	10.3	85%
Office	Human resources	4.0	3.3	3.3	10.5	88%
Office	Quality control	4.0	3.3	3.3	10.5	88%
Warehouse	Control at reception	3.5	3.3	3.0	9.8	81%
Warehouse	Warehouse organisation	4.0	3.3	3.3	10.5	88%
Warehouse	Physical storage conditions	4.0	3.5	3.3	10.8	90%
Warehouse	Management of the cold chain	3.5	3.5	3.8	10.8	90%
Warehouse	Stock Control	3.8	3.3	3.3	10.3	85%
Warehouse	Handling non conformity products	3.8	2.8	3.0	9.5	79%
Warehouse	Dispatch	4.0	3.0	3.3	10.3	85%
Warehouse	Transport	4.0	2.5	2.3	8.8	73%
	**Average of results from OFFICE settings**	**4.0**	**3.0**	**3.4**	**10.4**	**86%**
	**Average of results from WAREHOUSE settings**	**3.8**	**3.1**	**3.1**	**10.1**	**84%**
	**Overall average**	**3.9**	**3.1**	**3.2**	**10.2**	**85%**
		4: Higher score; 1: Lower score				

Table 9. Completeness, clarity and accuracy of QA information obtained for the four GSDP remote assessments

Ratings were set as:
- ‘Excellent’ when the average value was more than 3.5 out of 4.0 (out of 4.0)
- ‘Good’ when the average value was between 2.6 and 3.5 (out of 4.0)
- ‘Unsatisfactory’ when the average value was below 2.5 (out of 4.0).

The following findings can be highlighted:
- The **completeness**, i.e. the percentage of questions that were answered in the standard QA questionnaire, is set at an **excellent level** (overall average 3.9 out of 4.0).
- The **clarity**, i.e. the understandability of the information compiled in the standard QA questionnaire, is on average 3.1 out of 4.0, which corresponds to a **good level** of clarity.
- The **accuracy**, i.e. the reliability of the information compiled in the standard QA questionnaire, is on average assessed as 3.2 out of 4.0 (**good level** of accuracy).
- Overall, **the quality of data, which combines the results of completeness, clarity and accuracy, is excellent (10.2 out of 12.0, which is 85%).**
- The results show **very limited variation between the quality information assessed during *office work*** (desk-assessment of documentation: 86%) **and the one assessed during the *remote warehouse assessment*** (visit of premises: 84%). *Office work* covers the assessment of the QA system, documentation system, human resources, and quality control; while the *remote warehouse assessment* covers the assessment of the remaining aspects as for Table 10 below.

◎ **Quality of data by quality assurance activity** (Table 10)

		**Overall quality of data (percentage)**					
	**Quality assurance activity**	**distributor 1**	**distributor 2**	**distributor 3**	**distributor 4**	**average**	**standard deviation**
Desk assessment (office work)	QA system	75%	75%	100%	92%	**85%**	**13%**
	Documentation system	75%	92%	83%	92%	**85%**	**8%**
	Human resources	83%	83%	92%	92%	**88%**	**5%**
	Quality control	100%	83%	83%	83%	**88%**	**8%**
Remote assessment work	Control at reception	75%	83%	92%	75%	**81%**	**8%**
	Warehouse organisation	83%	83%	100%	83%	**88%**	**8%**
	Physical storage conditions	100%	75%	83%	100%	**90%**	**13%**
	Management of the cold chain	100%	100%	58%	100%	**90%**	**21%**
	Stock Control	83%	100%	100%	58%	**85%**	**20%**
	Handling non conformity products	67%	83%	92%	75%	**79%**	**11%**
	Dispatch	83%	92%	92%	75%	**85%**	**8%**
	Transport	75%	67%	83%	67%	**73%**	**8%**

Table 10. Quality of data by quality assurance activity

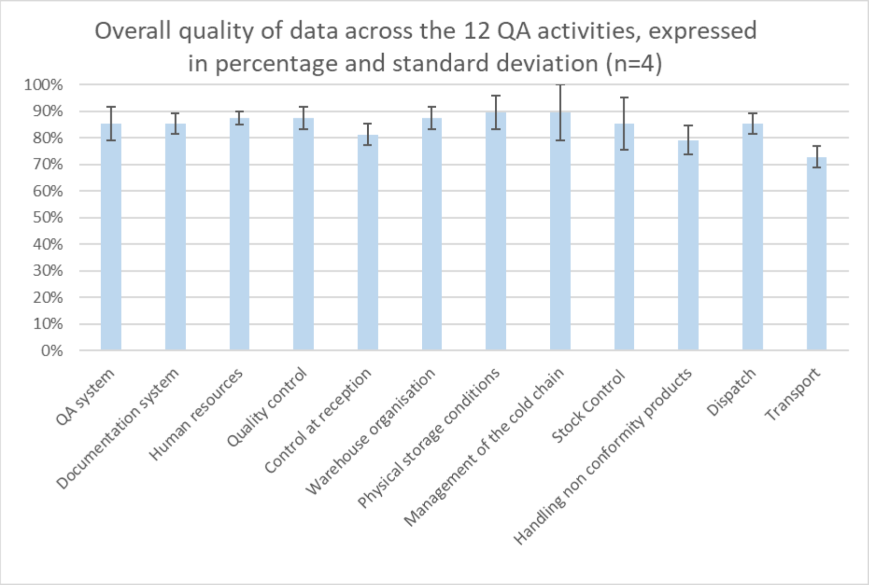

Figure 3. Overall quality of data across the 12 QA activities, expressed in percentage and standard deviation

The quality of data is relatively levelled across the 12 quality assurance activities (from 73% to 90%). The lowest values (i.e. less than 80%) are "handling non-conformity products" and "transport". The latter is likely to be due to the limited verification possibility of transport vehicles during the visit.

- **Qualification of the ICAFs**

The two ICAFs had different qualifications and previous experience with WHO GSDP. **The qualification of the ICAFs, however, did not seem to impact the quality of data, and therefore they do not seem a key determinant** for the success of the remote technical visit. However, it is clear that a basic knowledge of the GSDP, and an adequate comprehension of the assessment tool and the methodology, are key success factors for the remote work. Furthermore, both ICAF were carefully trained and provided with essential knowledge before the assessment, thus our finding here is conditional upon a careful and individualized training.

II. Overview of contextual limitations observed during the remote assessments

Type of limitation	Description of limitation	Occurrence	Comments
Technology related	Poor internet connection	4/4 (100%)	The study country has known poor telecommunications infrastructures.
	Audio conferencing breaks	4/4 (100%)	Short breaks occurred in all visits, leading to some repetitions.
	Video conferencing breaks	2/4 (50%)	In two warehouses, internet connection was very poor with a lot of video cuts. This was replaced by recording videos and pictures and transfer via WhatsApp. Also, the ICAFs were delegated some basic checks in the warehouse which could not be verified by the remote auditor.

Type of limitation	Description of limitation	Occurrence	Comments
Communication related	Languages barriers	1/4 (25%)	One distributor representative has an English level slightly below the others.
	QA wording barriers	2/4 (50%)	The QA concept is generally not well known in the study country, and the use of QA specific terminology was not always well understood by the distributor representatives.
Audit-related	Verification of documentation	4/4 (100%)	The verification of targeted documentation was sometimes quite challenging due to the local languages of the documents and the remote work. However, as quality systems were generally poor in all four distributors, there were relatively few documents to verify: some documents (SOPs and records required in a QA system) were simply not available. The ICAFs played sometimes a translator role.

Table 11. Overview of contextual limitations observed during the remote assessments

III. Good practices needed for remote assessments

Topics	Good practices identified
**Preparation**	List of documents to be sent prior to the assessment (at least 5 working days if possible)
	Selection of distributors with good level of English/French/others languages, in line with auditor language proficiency
**Data collection tools**	Templates with two columns: the first one for the QUAMED expert, and the second one for the ICAF (each section should be filled out independently, and copied/pasted afterwards)
	Several printouts of the tool should be available in advance, to be used by the ICAFs, but also given, if necessary, to the distributor to ease the communication
**ICAF training**	Briefing about the way of working, the auditing attitude, the sequences of a technical visit, and the essentials of GSDP.
	Upfront sharing of SOPs and tools
	Tailoring the training based on each person's previous knowledge and expertise
**Technology**	Good smartphone available, with good video and photo definition, fully charged, and with sufficient credit for internet data. Tablets may also be used.
	Back-up smartphone, fully charged also
	Laptop with video conferencing software installed, fully charged
**Communication**	It could be helpful to have smartphone video filmed by a third person, which would allow the auditor/PI to see both the ICAF and the distributor representative, during the visit of the warehouse.
**Timescale**	Slightly more time to be planned than an on-site technical visit

Table 12. Good practices needed for remote assessments

IV. Feedbacks from ICAFs

The feedbacks from ICAFs are presented in the **Annex 4.** They reported that:

- They were both very interested in participating in the study;

- The preparation phase was adequate;
- The tools used were appropriate (one suggested to have the questionnaire translated in the local language);
- The main challenges were mostly linked to internet and smartphone technology.

Furthermore, they both communicated to the PI their interest in quality assurance work, and they requested training materials. This had not been anticipated or foreseen when writing the protocol, but it indicates that such remote assessments can create the opportunity to raise awareness among (international and local) NGOs staff members about the needs to assess distributors on their quality assurance systems, and to build local capacities.

V. Data analysis of sourcing assessment

Data provided by the assessed distributors about the products they supplywere found to be complete and accurate. This is not surprising, as the methods are identical between an on-site and a remote assessment. When a sourcing assessment is carried out during an on-site Local Market Assessment, the data are extracted from the product list provided by the company. The QUAMED expert then inserts information from the list in the QUAMED template (refer to Figure 2), and the QUAMED technical coordinator completes the template (level of GMP according to QUAMED standards) based on independent information available in the database of QUAMED. The system used during the remote work is similar as the one carried out during an on-site visit. The same tasks were assigned to the QUAMED expert and the QUAMED technical coordinator.

As a conclusion, we did not detect any difference in the set-up and the implementation of sourcing assessment, either carried out during an on-site local market assessment or as remote work, as long as source data are transmitted by a trusted person. This evaluation only concerns the feasibility of the assessment, and not the quality of the products supplied by these distributors.

5. Discussion

- **Technical feasibility of GSDP technical visit**

The completeness, i.e. the percentage of questions related to specific QA activities that were answered in the standard questionnaire, looked excellent (average 3.9 out of 4.0 across the 4 distributors in our sample). The clarity (i.e. understandability) of the answers (average 3.1 out of 4.0) and its accuracy (average 3.2 out of 4.0) were good. Overall, the “quality of data”, which combines completeness, clarity and accuracy, was good (10.2 out of 12.0, which is 85% of the best possible score). We also noted low variations across distributors (range 83-88%), across activities (range 73%-90%), and between data obtained from “office” (desk-assessment of documentation: 86%) versus “warehouse” (visit of premises: 84%).

This preliminary work suggests that the intrinsic limitations of remote assessments, e.g. unstable internet connection, languages barrier and impossibility for auditors to personally verify premises and documents, can be mitigated by adopting adequate Good Practices. Language skills at the audited suppliers should be acceptable to ensure adequate communication. We believe that a careful implementation of the good practices listed in the Results section can ensure the feasibility and reliability of remote assessments. These good practices are related to the different steps of a remote GSDP assessment: preparation, tools, training, and technology.

However, our findings may be biased by the fact that we inspected distributors located in a country with wear regulatory supervisions, thus with poor practices and succinct quality systems. Findings could be different when assessing a distributor with a complete QA system in place (more documentation to be reviewed) or in a better-regulated country. Therefore, our findings cannot be extrapolated to very different contexts.

- **Technical feasibility of sourcing assessment**

As per the set-up and the results, there is no substantial difference in the set-up and the implementation of sourcing assessment carried out within the scope of an on-site local market assessment or as remote work,

provided that source data may be provided by a trusted person on site. Therefore, remote sourcing assessments, if supported by a well-trained ICAF:
- Are feasible remotely;
- Do not imply additional risks compared to an on-site assessment;
- Do not require a change in the standard procedure, or in the tool used.

◎ A QUAMED-specific reflection: inclusion of results of remote GSDP technical visit in the QUAMED database: Approach and determinants

The inclusion of remote technical visit results (including the ones from this pilot study) in the QUAMED database means that the information could be used by the QUAMED members for decision -making on local purchase. Currently, we recommend to consider inclusion case-by-case, based on a patient-centred risk-based approach:

Recommendation from the remote technical visit	Inclusion of results in QUAMED database	Rationale
Supplier not recommended (GSDP rating less than 60%)	Yes	Minimal risk (and no risk for patients)
Supplier recommended, or conditionally recommended	Subject to validation of the results by the QUAMED technical committee	Risk-based approach linked with the quality of data, the results, and the technical visit limitations. A wrong recommendation would be harmful to patients

Table 13. Inclusion of results of remote GSDP assessments in the QUAMED database: Approach and determinants

Remote technical visits should be clearly indicated in the QUAMED database, and the report should begin with a well-visible cautionary statement.

◎ Others impacts

- Environmental impact

For this pilot study, the savings in term of CO_2 emission is equivalent to approximately 1.2 tons of CO_2. This takes into consideration the “saved” flights.

Even if the on-site technical visits remain the gold standards, perhaps remote technical visits could be considered for following up on initial on-site technical visits, CAPA etc. Usually this is not done due to lack of budget for follow-up, thus the remote technical visits would improve the ‘gold standard’. Similarly, the remote technical visits could be used for a pre-screening of suppliers to be included in LMA, which is not possible in-person.

- ‘Customer’ satisfaction

QUAMED members may be reluctant to trust the findings of remote technical visits, even if it will be always clearly stated that the on-site technical visits remains the gold standards. On a more positive note, the response time to a specific need (time between the need expression and the availability of the results) may be quicker using remote work, in particular for assessments of limited number of distributors. This may be a crucial aspect in some humanitarian emergency settings, and an initial remote technical visits could be later completed by an on-site one.

- Financial aspects

On-site assessments are costly in term of accommodation, visa fees, per diem, and transport, and a remote assessment allows many savings, while requiring little extra expenditure (internet access fees, electricity).

Additionally, as noted above, an online pre-screening could avoid unnecessary visits at suppliers which are of very poor quality, allowing more savings in terms of time and money.

Remote work for others types of assessments

At this stage, it seems too risky to propose all types of assessments (GMP and GDP audits, technical visits) for remote assessment. The following table may be used to structure a broader reflection on the possibility to expand remote assessments (based, also in this case, on a patient-centered risk assessment):

Type of assessment		Primary recommendations
**GMP audits**	Full audit	Not recommended (risks of "false positives" too high)
	Partial audit	May be feasible remotely, but to be assessed on a case-by-case basis (for instance linked to documentation review, or for an initial prescreening of sites that are candidate for a full onsite audit)
	Follow-up audit	Depending on the criticality of observations and their verification in remote settings, the feasibility could be further explored
**MQAS/GSDP audits**	Full audits	Not recommended (risks of "false positives" too high)
	Partial audit	May be feasible remotely, but to be assessed on a case-by-case basis (for instance, for audits covering only prequalification aspects, or for an initial prescreening of candidates for a full onsite audit)
	Follow up audit	Depending on the criticality of observations and their verification in remote settings, the feasibility could be further explored
**GMP technical visits***	N/A	Not recommended (risks of "false positives" too high)

** GMP technical visit are short visit with limited scope compared to a full GMP audit*

6. Conclusion

Our findings suggest that a well-conducted remote assessment of pharmaceutical distributors may provide data of acceptable quality, allowing to assess their GSDP-compliance and to make temporary decisions about licensing (regulators) or purchasing (international organizations, NGOs). However, policy-makers should read the results of remote assessments in light of their intrinsic limitations, and they should aim to reinstall in-person assessments as soon as possible after the end of a crisis. The use of remote assessments could be separately considered for either pre-screening of candidates for on-site assessments, or targeted follow-up of on-site assessment.

Our findings should be confirmed in other contexts (e.g. in better-regulated countries) and they cannot be extrapolated to others types of audits ("Good Manufacturing Practices" or "Model Quality Assurance. System for Procurement Agencies").

Extended scope of work for QUAMED

The use of remote work should always be linked with a patient-centered risk-based approach. It is suggested that QUAMED may use remote work for the following topics:

- Screening prior to MQAS audits or GSDP audits, or even GSDP/GMP technical visits: the exercise could be very useful to avoid waste of time on the field when the assessment type is not well chosen. It may also be cost-effective for NGOs.
- Follow-up work on CAPA
- Mock audit: This may be used for the coming QUAMED QCP (Quality Certification Program).
- Remote product source assessment: this could be a new service offered by QUAMED. However, the methodology should be further developed, and the added value of this service would be conditional on availability of a very comprehensive product quality ("GMP") database.

Next research steps

The methodology used in this study could be applied to collect some remote data, from some distributors that will be assessed on-site in the next months. This would allow a more direct comparison vs the gold standard. Furthermore, more remote pilot technical visits may be conducted in other countries, with other ICAFs, to check the replicability of our findings. During this phase other tools, such as smart glasses or screen sharing features, could be tested.

7. Annexes

Annex 1. Research protocol

Annex 2. Presentation of the tool used for remote GSDP technical visit (adapted from the tool used for local market assessment)

Annex 3. Results from the individual technical visits

Annex 4. Results from the feedback forms completed by the ICAFs after each individual technical visits

Annex 1: Research protocol (embedded)

201102 QUAMED
ITM research protoco

Annex 2: Presentation of the tool used for remote GSDP technical visit (adapted from the tool used for local market assessment)

COLOR CODE FOR ASSESSMENT TEAM	
(turquoise)	Questions may be **fully managed by in-country assessment facilitator**, while the main assessor is on video/audio conference and may request clarifications
(light blue)	Questions are mostly **managed by the QUAMED expert** through IT communication tools (office).
(purple)	Questions are **managed by either the QUAMED expert or ICAF during the visits**

Colour code system to ease the identification of each task between the ICAF and the QUAMEED expert

i. Technical visit information		
1	Name of the company:	
2	Website:	
3	Key contact details # 1	Name:<br>Position:<br>Email :<br>Phone:
4	Key contact details # 2 (if required)	Name:<br>Position:<br>Email :<br>Phone:

Clear identification in the questionnaire of the lead assessor using the colour code system

Human Resources		Rating	Justifi[illegible]
3.1	*Please describe how the Quality Assurance departement is organised (if any).*	*N/A*	
3.2	*Is an organization chart available? Is it up to date and signed? Does the chart reflect the independence of QA from operations?*	0%	
ICAF	*Get the organization chart*	N/A	
3.3	*How many pharmacists are currently employed by the company?*	0%	
3.4	*Is there a position with specific QA responsibilities?*	0%	
3.5	*Are written job descriptions available for key positions?*	0%	
ICAF	*Get the job descrption of the QA officer / pharmacist in charge*	N/A	
3.6	*Does the company have a training program? Are the key personnel trained regarding its tasks?*	0%	
ICAF	*Get a example of training records for the warehouse staff*	N/A	
Global rating (percentage of compliance)		***0%***	*automatic calculation*

Description of additional tasks to be done during the visit by the ICAF

LIMITATIONS OF THE TECHNICAL VISIT DUE TO THE REMOTE CONTEXT

Report of all limitations that occurred during the technical visit

Annex 3. Results from the individual technical visits

Distributor 1, assessment on 2nd November 2020 by A. Bourasseau

	Information	Review of data / information collected: Completeness	Clarity	Accuracy	Overall quality of data		Comments on (any) significant variations vs previous assessment	Limitations	Comments
	Instructions on how to report the information	*The info obtained was complete, i.e. all questions were answered*	*The info obtained was clear, i.e. all questions were answered in an understandable way*	*The info obtained seemed accurate, i.e. the auditor did not have the impression that questions were answered without checking on sourced data / documents*	*A combination of completeness, clarity, and accuracy*		*No previous assessment*	*List limitations by category: due to the audio or video connection; due to the link-person on site; etc.*	*For instance: perception that some information was not disclosed on purpose (intention to deceive?); comments on the attitude on the audited team; comments on the quality of the audio and video connection; comments on the skills/role of the link peron on site;*
Office	QA system	4	2	3	9	75%	N/A	Some short connexion cuts during the assessment	Use of whatsapp (video conferencing)
	Documentation system	4	2	3	9	75%	N/A		
	Human resources	4	3	3	10	83%	N/A		
	Quality control	4	4	4	12	100%	N/A		
Warehouse	Control at reception	3	3	3	9	75%	N/A	Some short connexion cuts during the assessment<br>Quality of video sometimes not very clear due to limited connexions, but limited in time	Use of whatsapp (video conferencing)
	Warehouse organisation	4	3	3	10	83%	N/A		
	Physical storage conditions	4	4	4	12	100%	N/A		
	Management of the cold chain	4	4	4	12	100%	N/A		
	Stock Control	4	3	3	10	83%	N/A		
	Handling non conformity products	3	2	3	8	67%	N/A		
	Dispatch	4	3	3	10	83%	N/A		
	Transport	4	3	2	9	75%	N/A		
		4: Higher score; 1: Lower score			10,0	83%			

Distributor 2, assessment on 4th November 2020 by A. Bourasseau

	Information	Review of data / information collected: Completeness	Clarity	Accuracy	Overall quality of data		Comments on (any) significant variations vs previous assessment	Limitations	Comments
	Instructions on how to report the information	*The info obtained was complete, i.e. all questions were answered*	*The info obtained was clear, i.e. all questions were answered in an understandable way*	*The info obtained seemed accurate, i.e. the auditor did not have the impression that questions were answered without checking on sourced data / documents*	*A combination of completeness, clarity, and accuracy*		*Previous QUAMED visit on 17/07/2020. It should be noted that the GDP technical visit tool has changed between the two visits.*	*List limitations by category: due to the audio or video connection; due to the link-person on site; etc.*	*For instance: perception that some information was not disclosed on purpose (intention to deceive?); comments on the attitude on the audited team; comments on the quality of the audio and video connection; comments on the skills/role of the link peron on site;*
Office	QA system	4	2	3	9	75%	No comments	Some short connexion cuts during the assessment	Use of whatsapp (video conferencing).<br>English level of the distributor director is average -> this lead to sometimes difficulties to understand each other
	Documentation system	4	3	4	11	92%	No comments		
	Human resources	4	3	3	10	83%	Job descriptions are now available		
	Quality control	4	3	3	10	83%	No comments		
Warehouse	Control at reception	4	3	3	10	83%	No comments	Network problem during the visit of the warehouse due to poor connexion: The ICAF did the visit on tis own with the questionnaire and sent picture, video and records to the QUAMED auditor	Use of whatsapp (video conferencing)<br>English level of the distributor director is average -> this lead to sometimes difficulties to understand each other
	Warehouse organisation	4	3	3	10	83%	No comments		
	Physical storage conditions	4	3	2	9	75%	Temperature records are now available		
	Management of the cold chain	4	4	4	12	100%	No comments		
	Stock Control	4	4	4	12	100%	Computerised system is now in place		
	Handling non conformity products	4	3	3	10	83%	No comments		
	Dispatch	4	3	4	11	92%	Batch traceability is now ensured		
	Transport	4	2	2	8	67%	No comments		
		4: Higher score; 1: Lower score			10,2	85%	GDP score of the first visit : **27%***<br>GDP score of this visit: **28%***	** rating system has however changed between both assessments*	

Distributor 3, assessment on 5th November 2020 by A. Bourasseau

		Review of data / information collected					Comments on (any) significant variations vs previous assessment	Limitations	Comments
	Information	Completeness	Clarity	Accuracy	Overall quality of data				
	Instructions on how to report the information	*The info obtained was complete, i.e. all questions were answered*	*The info obtained was clear, i.e. all questions were answered in an understandable way*	*The info obtained seemed accurate, i.e. the auditor did not have the impression that questions were answered without checking on sourced data / documents*	*A combination of completeness, clarity, and accuracy*		*Previous QUAMED visit on 17/07/2020. It should be noted that the GDP technical visit tool has changed between the two visits.*	*List limitations by category: due to the audio or video connection; due to the link-person on site; etc.*	*For instance: perception that some information was not disclosed on purpose (intention to deceive?); comments on the attitude on the audited team; comments on the quality of the audio and video connection; comments on the skills/role of the link peron on site;*
Office	QA system	4	4	4	12	100%	SOPs have been drafted now	Some short connexion cuts during the assessment	Use of whatsapp (video conferencing)<br>The director has very good english, which ease the communication
	Documentation system	4	3	3	10	83%	SOPs have been drafted now		
	Human resources	4	4	3	11	92%	Organisation chart and job descriptions are now available & OK		
	Quality control	4	3	3	10	83%	No comments		
Warehouse	Control at reception	3	4	4	11	92%	No comments	Some short connexion cuts during the assessment<br>Camera from the ICAF smartphone was not working -> but up with the suppliers director smartphone	Use of whatsapp (video conferencing)<br>The director has very good english, which ease the communication
	Warehouse organisation	4	4	4	12	100%	Quarantine area is now available		
	Physical storage conditions	4	3	3	10	83%	No comments		
	Management of the cold chain	2	2	3	7	58%	No comments		
	Stock Control	4	4	4	12	100%	No comments		
	Handling non conformity products	4	4	3	11	92%	No comments		
	Dispatch	4	4	3	11	92%	No comments		
	Transport	4	3	3	10	83%	No comments		
		4: Higher score; 1: Lower score			10,6	88%	GDP score of the first visit : **35%***<br>GDP score of this visit: **44%***		

Distributor 4, assessment on 23rd November 2020 by A. Bourasseau

		Review of data / information collected					Comments on (any) significant variations vs previous assessment	Limitations	Comments
	Information	Completeness	Clarity	Accuracy	Overall quality of data				
	Instructions on how to report the information	*The info obtained was complete, i.e. all questions were answered*	*The info obtained was clear, i.e. all questions were answered in an understandable way*	*The info obtained seemed accurate, i.e. the auditor did not have the impression that questions were answered without checking on sourced data / documents*	*A combination of completeness, clarity, and accuracy*		*No previous assessment*	*List limitations by category: due to the audio or video connection; due to the link-person on site; etc.*	*For instance: perception that some information was not disclosed on purpose (intention to deceive?); comments on the attitude on the audited team; comments on the quality of the audio and video connection; comments on the skills/role of the link peron on site;*
Office	QA system	4	3	4	11	92%	N/A	* Sounds from the phone not so good	Use of whatsapp video<br>Good English level
	Documentation system	4	3	4	11	92%	N/A		
	Human resources	4	3	4	11	92%	N/A		
	Quality control	4	3	3	10	83%	N/A		
Warehouse	Control at reception	4	3	2	9	75%	N/A	* Sounds from the phone not so good<br>* Numerous cuts of the video conferencing in the warehouse (no network from the middle of the warehouse) -> The visit was shortened, but a whatsapp call was organised the following day	Use of whatsapp video<br>Good English level<br>Additionnal information collected by email and additional Whatsapp call after the visit
	Warehouse organisation	4	3	3	10	83%	N/A		
	Physical storage conditions	4	4	4	12	100%	N/A		
	Management of the cold chain	4	4	4	12	100%	N/A		
	Stock Control	3	2	2	7	58%	N/A		
	Handling non conformity products	4	2	3	9	75%	N/A		
	Dispatch	4	2	3	9	75%	N/A		
	Transport	4	2	2	8	67%	N/A		
		4: Higher score; 1: Lower score			9,9	83%			

Annex 4. Results from the feedback forms completed by the ICAFs after each individual assessment

**Do you think the preparation of the GSDP technical visit was sufficient?**
• Yes, it was sufficient, the visit included complete package of technical questions. It covered all the areas were intended for the assessment and relevant data were gathered.<br>• Yes, it was completely sufficient
**If not, what could have been improved/done differently?**
**What are the elements that were successful during the assessment?**
• **Related to the I.T. communication tools**
• Smart phone and video call through WhatsApp
• **Related to the methodology used**
• A participatory and client oriented methodology was used during this assessment where both the assessor and the client could feel free to share the information asked by the assessor. Structured interview and open questions were helpful during the assessment process.<br>• Yeah it was good, but it was the first time that we had remote site visit. there is always some problems at the beginning.
• **Related to the tools used for the technical visit**
• A useful questionnaire is used during the assessment process which could help the assessor to structure its interview process and record the answers , finding and observations.<br>• Our only problem was internet and connection problem
• **Related to the communication between the QUAMED expert and yourself?**
• Communication was in a very good manner and the distributer feels very comfortable and because of good communications, they provide us all the required document which had and answer our all questions.<br>• When ever asked for any help he was ready, the communication was perfect.
• **Related to other elements not mentioned before**
• N/A
**What could have been improved during the assessment?**
• **Related to the I.T. communication tools**
• The internet connection was poor and frequently were cut that disturbed our work during the assessment process.
• **Related to the methodology used**
• The methodology has been improved
• **Related to the tools used for the technical visit**

• The tool used during the assessment was perfect, and it covered all the areas required for this assessment.<br>• Let's have the assessment tools shorter next time with less questions for the companies.
• **Related to the communication between the QUAMED expert and yourself?**
• The communication between myself and the QUAMED expert improved my interest and knowledge.<br>• The communication was perfect
• **Related to other elements not mentioned before**
• It's better to have the assessment tools translation in local languages as well for the companies.
**What are the limitations of factors?**
• Distance assessment is always a challenge but we tried our best to decrease the level of challenges instead we tried our best to do our assessment get done. As I said connection problem was one of the biggest challenges some times during the assessment we lost the connections.<br>• The only limitation was the weak internet connection that always cut and disturbed our communications.
**Did you identify some good practices that could be used for other assessments?**
• Yes it was a good practices and it also helped the companies to fine the gap and the shortages they had to solve it in the future.<br>• Generally, it was my first time to be a part of this kind of assessment it was overall good practice for me and I have learned a lot of things that I can use for other assessments, specifically the manner of communication in such kind of assessment the way how to ask the distributor, knowing what standards should be applied by distributer were good practices from me.
**Do you have any other comments you would like to share?**
• No thanks from QUAMED and team it was very useful technical site visit and assessment we learned a lot and it was a good experience.<br>• I don't have any comment, just want to mention that it was a very useful and successful assessment and I would like to be a part of future assessments.

References

[i] African Medicines Regulatory Harmonization Initiative (AMRHI): a WHO concept paper. WHO drug Information, vol. 22, no. 3, pp. 182-190

[ii] Ravinetto R, Roosen T, Dujardin C. The Belgian commitment to pharmaceutical quality: a model policy to improve quality assurance of medicines available through humanitarian and development programs. Journal of Pharmaceutical Policy and Practice 2018; 11:12: 1-5

[iii] World Health Organization. Model quality assurance system for procurement agencies. In: Annex III of the WHO technical report series 986: WHO Expert Committee on Specifications for Pharmaceutical Preparations, forty-eighth report. World Health Organization, 2014

[iv] World Health Organization, Annex 7: WHO 'Good storage and distribution practices for medical products', WHO Technical Report Series, No. 1025, 2020

[v] World Health Organization, Annex 2: WHO good manufacturing practices for pharmaceutical products: main principles' WHO Technical Report Series No. 986, 2014

[vi] European Medicines Agency. Guidance related to GMP/GDP and PMF distant assessments. EMA/335293/2020. https://www.ema.europa.eu/en/documents/scientific-guideline/guidance-related-gmp/gdp-pmf-distant-assessments_en.pdf. Accessed on 15th December 2020

[vii] Medical Device and Single Audit Program Transmittal Number: 2020-07. https://www.fda.gov/media/136441/download. Accessed on 13th November 2020

[viii] European Medicines Agency, Guidance on remote GCP inspections during the COVID19 pandemic, EMA/INS/GCP/162006/202018, May 2020. https://www.ema.europa.eu/en/documents/regulatory-procedural-guideline/guidance-remote-gcp-inspections-during-covid-19-pandemic_en.pdf. Accessed on 7th January 2021

[ix] Medical Device Coordination Group Document, MDCG 2020-4 - European Commission, MDCG 2020-4 https://ec.europa.eu/health/sites/health/files/md_sector/docs/md_mdcg_2020_4_nb_audits_covid-19_en.pdf. Accessed on 13th November 2020

[x] Medical Device Coordination Group Document Remote Auditing Pilot Program. MDSAP AU P0036.001 https://www.fda.gov/media/134229/download Accessed on 13th November 2020

[xi] https://www.pharmaceuticalonline.com/doc/remote-auditing-best-practices-for-gmp-compliance-0001. Accessed on 23rd November 2020

[xii] https://www.freyrsolutions.com/remote-and-virtual-audit. Accessed on 23rd November 2020
